# *MSH6* germline mutations in early-onset colorectal cancer patients without family history of the disease

**DOI:** 10.1038/sj.bjc.6603318

**Published:** 2006-08-29

**Authors:** C Pinto, I Veiga, M Pinheiro, B Mesquita, C Jeronimo, O Sousa, M Fragoso, L Santos, L Moreira-Dias, M Baptista, C Lopes, S Castedo, M R Teixeira

**Affiliations:** 1 Department of Genetics, Portuguese Oncology Institute, Porto, Portugal; 2 Department of Radiotherapy, Portuguese Oncology Institute, Porto, Portugal; 3 Department of Oncology, Portuguese Oncology Institute, Porto, Portugal; 4 Department of Surgery, Portuguese Oncology Institute, Porto, Portugal; 5 Department of Gastroenterology, Portuguese Oncology Institute, Porto, Portugal; 6 Department of Surgery B, S. João Hospital, Porto, Portugal; 7 Department of Pathology, Portuguese Oncology Institute, Porto, Portugal

**Keywords:** *MSH6*, germline mutations, early-onset colorectal cancer

## Abstract

Germline *MLH1* and *MSH2* mutations are scarce in young colorectal cancer patients with negative family history of the disease. To evaluate the contribution of germline *MSH6* mutations to early-onset colorectal cancer, we have analysed peripheral blood of 38 patients diagnosed with this disease before 45 years of age and who presented no family history of hereditary nonpolyposis colorectal cancer-related cancers. Blood samples from 108 healthy volunteers were analysed for those genetic alterations suspected to affect the function of MSH6. Of the seven (18.4%) *MSH6* alterations found, we have identified three novel germline mutations, one 8 bp deletion leading to a truncated protein and two missense mutations resulting in the substitution of amino acids belonging to different polarity groups. High-frequency microsatellite instability was found in the patient with the *MSH6* deletion, but not in the other 27 carcinomas analysed. No *MLH1* promoter methylation was detected in tumour tissue. Our findings suggest that germline *MSH6* mutations contribute to a subset of early-onset colorectal cancer. Further studies are warranted to understand the genetic and environmental factors responsible for the variable penetration of *MSH6* germline mutations, as well as to identify other causes of early-onset colorectal cancer.

Germline mutations in mismatch repair (MMR) genes (mostly in *MLH1* and *MSH2*) are associated with hereditary nonpolyposis colorectal cancer (HNPCC), a highly penetrant autosomal-dominant syndrome characterised by several affected individuals with colorectal cancer (CRC) or extracolonic tumours of the endometrium, stomach, small bowel, ureter, renal pelvis, ovary, and hepatobiliary tract (Lynch and de la Chapelle, 2003). Hereditary nonpolyposis colorectal cancer accounts for about 3–5% of all CRC, including a large proportion of those with a young age of diagnosis, representing the most common hereditary colon cancer syndrome (Petersen *et al*, 1999; Umar *et al*, 2004). Hereditary nonpolyposis colorectal cancer carriers are usually heterozygous for the mutant allele, retaining a functional copy of the gene that is apparently sufficient for DNA repair (Parsons *et al*, 1993). A subsequent somatic mutation, leading to inactivation of the wild-type allele, results in a mutator cancer phenotype named ‘high-frequency microsatellite instability’ (MSI-H), characterised by a high rate of base substitutions, as well as small insertions and deletions in mono- and dinucleotide repeats (Ionov *et al*, 1993). High-frequency microsatellite instability phenotype has also been reported in sporadic CRC (10–15%), but somatic mutations in MMR genes are rare in these cases (Peltomaki *et al*, 1993; Kuismanen *et al*, 2000; Kamory *et al*, 2003). *MLH1* promoter region methylation, leading to silencing of this gene, is an alternative mechanism to mutation underlying MSI-H in sporadic CRC (Toyota *et al*, 1999). Genetic testing for hereditary predisposition is therefore critical for effective management of suspected HNPCC kindreds (de la Chapelle, 2004; Lynch *et al*, 2004; Hampel *et al*, 2005).

The identification of hereditary predisposition is not always easy based on clinical and familial data. Some CRC patients are diagnosed at a very young age, but do not show a family history of cancer indicative of HNPCC. The relative contribution of environmental and genetic factors for the development of the disease in this subset of patients is unknown, but previous investigations have shown that germline mutations in *MLH1* and *MSH2* genes are scarce (Southey *et al*, 2005). *MSH6* germline mutations have been mostly observed in atypical HNPCC families presenting a weaker family history, possibly caused by lower penetrance of mutations in this gene (Hendriks *et al*, 2004; Plaschke *et al*, 2004), and tumours in these families may be ‘microsatellite stable’, may present ‘low-frequency microsatellite instability’ (MSI-L), or may be MSI-H (Wu *et al*, 1999; Plaschke *et al*, 2000; Berends *et al*, 2002). We therefore aimed to evaluate the contribution of germline *MSH6* mutations for early-onset CRC in patients without a family history of HNPCC-related cancer.

## MATERIALS AND METHODS

### Patients, samples, and DNA extraction

After written informed consent, DNA was isolated from peripheral blood samples of 38 individuals who developed CRC before the age of 45 years and had negative family history of the disease (or other HNPCC-associated cancers; Table 1), using the salt–chloroform extraction method (Müllenbach *et al*, 1989). DNA extraction was also performed from paraffin-embedded tumours available from 28 of the patients (Lungu *et al*, 1992), as well as from peripheral blood of 108 healthy volunteers. The study was approved by the Institution Review Board.

### Screening for germline *MSH6* mutations

DNA from the 38 CRC patients were screened for *MSH6* mutations by denaturing gradient gel electrophoresis (DGGE) using primers and conditions described previously (Wu *et al*, 1999). Exon 1 and the acceptor splice site of exon 10, as well as samples with abnormal DGGE patterns, were analysed by direct sequencing in an *ABI PRISM* 310 automatic sequencer using Big Dye Terminator Chemistry (Applied Biosystems, Foster City, CA, USA), according to the manufacturer's recommendations. Whenever necessary, exon 7 was resequenced using different set of primers to exclude or confirm the presence of a polymorphism at the initial primer-annealing site (Kolodner *et al*, 1999).

### MSI analysis

Of the 38 cases, DNA from 28 paraffin-embedded tumours and paired lymphocyte samples were evaluated for MSI. We analysed the Bethesda marker panel (Boland *et al*, 1998), which includes two mononucleotide repeats (BAT25 and BAT26) and three dinucleotide repeats (D2S123, D5S346, and D17S250). Another three mononucleotide repeat markers (BAT34C4, BAT-RII, and BAX) were also analysed, as MSI in *MSH6* carriers has been mainly observed in this type of markers (Verma *et al*, 1999; Wijnen *et al*, 1999). DNA was amplified by PCR using fluorescence-labelled 5′ primers, as described previously (Oliveira *et al*, 1998; Zhou *et al*, 1998; Pyatt *et al*, 1999; Loukola *et al*, 2001), and analysed in an *ABI PRISM* 310 automatic sequencer. Cases were considered MSI-H when ⩾30% of the markers were positive. The MSI-H case (see below) was routinely analysed for *MLH1* and *MSH2* germline mutations, using multiplex ligation-dependent probe amplification (MRC-Holland, Amsterdam), DGGE, and direct sequencing with primers and conditions described previously (Wu *et al*, 1997, 1998), and no pathogenic mutation was detected in these two genes.

### *MLH1* promoter methylation analysis

*MLH1* promoter methylation status was evaluated in MSI-positive cases (both MSI-H and MSI-L), using methylation-specific PCR with primers and conditions described by Fleisher *et al* (2001).

## RESULTS

Seven of the 38 patients (7/38; 18.4%) presented germline *MSH6* changes (Table 2), six of them not described previously. One patient had the deletion c.3558_3565delTGAAAGTA (p.G1186fsX1190), two patients presented the missense mutations c.2633T>C (p.V878A) and c.3961A>G (p.R1321G), two patients had the silent mutations c.2272C>T (p.L758L) and c.2319C>A (p.L773L), one patient showed the intronic alteration c.3439-16C>T, and another patient had a silent mutation and a missense mutation, c.[648A>T; 649G>T] (p.[T216T; D217Y]), in the same allele (Table 2).

**Figure 1:**
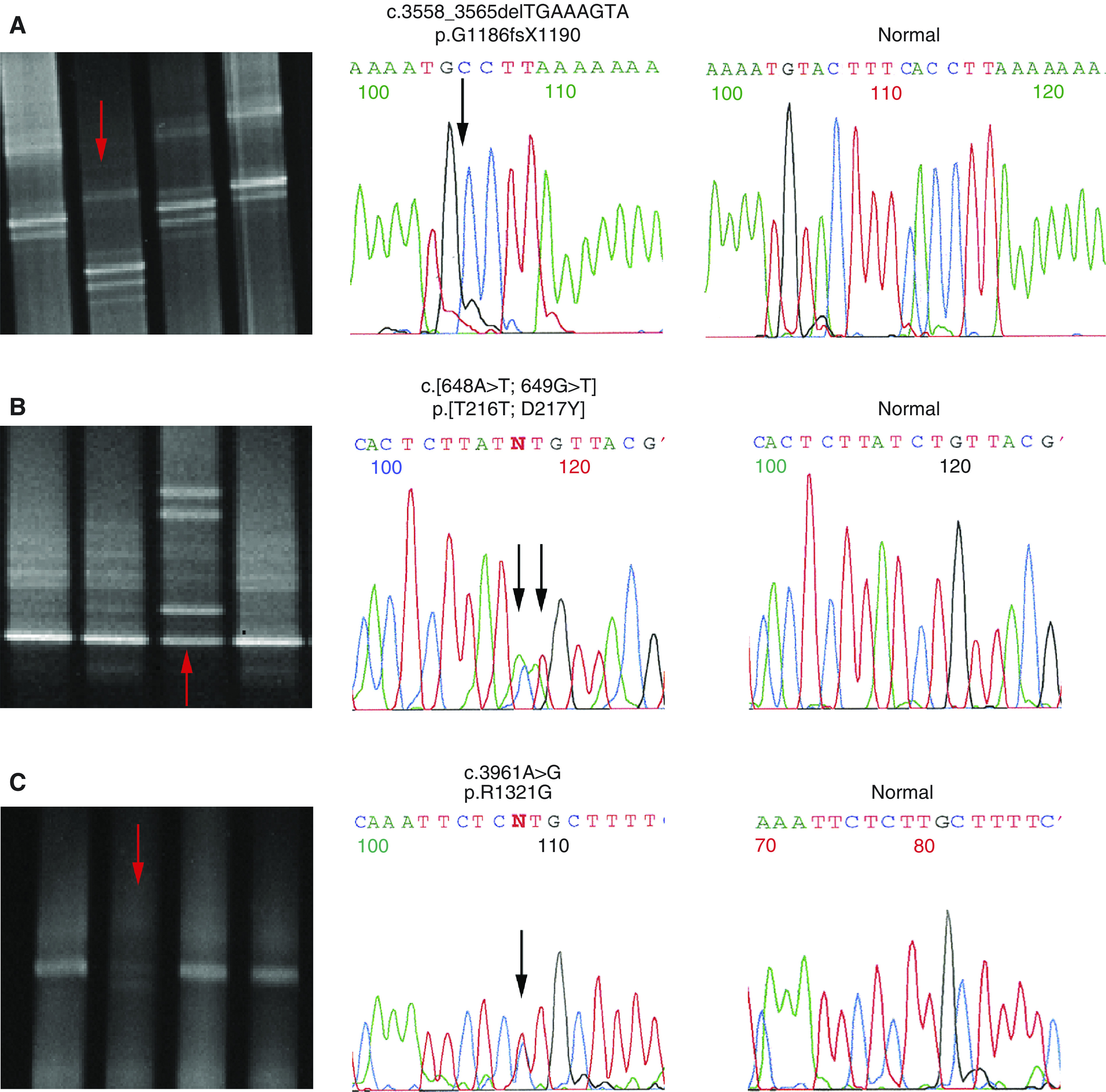
Possible disease-causing *MSH6* mutations (arrows) identified in early-onset CRC patients. Denaturing gradient gel electrophoresis patterns and the nucleotide sequences (reverse) are shown for cases 2 (**A**), 37 (**B**), and 14 (**C**). Negative control sequences are shown to the right.

**Figure 2:**
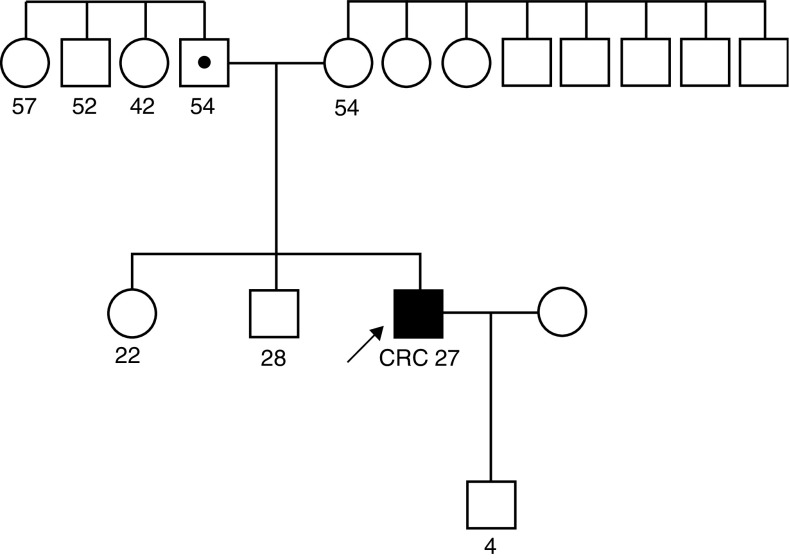
Family pedigree of patient nr. 2. Filled symbol (arrowed) represents the affected proband harbouring the heterozygous mutation p.G1186fsX1190 and the symbol with a black circle represents a carrier. Numbers below the symbols indicate age at cancer diagnosis or age at last observation if unaffected. CRC: colorectal cancer.

In cases in which changes were suspected to affect the function of MSH6, namely, the deletion, the missense, and the intronic alterations, we looked for these nucleotide changes in blood samples from 108 healthy volunteers. The p.G1186fsX1190 and the c.3439-16C>T alterations were detected once and p.V878A in another two controls (Table 2). The p.G1186fsX1190 mutation was initially detected in apparent homozygosity (Figure 1) in the CRC patient and in the control using primers described by Wu *et al* (1999). Direct sequencing with additional primers demonstrated that these cases were in fact heterozygous for this mutation and that an insertion polymorphism in intron 7 located in the primer-annealing region had not allowed the amplification of one allele with the initial set of primers (data not shown). The p.G1186fsX1190 mutation was subsequently identified in the healthy 54-year-old father of the proband (Figure 2).

High-frequency microsatellite instability and MSI-L was found in one and two of the 28 tumours, respectively. The MSI-H patient was positive only for the mononucleotide markers BAT25, BAT26, and BATRII and presented the p.G1186fsX1190 mutation. The two MSI-L patients were positive only for one marker and had no germline mutations in the *MSH6* gene. None of these three cases showed *MLH1* promoter hypermethylation in the tumour (data not shown).

## DISCUSSION

Molecular characterisation of early-onset CRC is important to clarify whether this clinical feature is caused by dominant germline mutations with variable penetrance or arising *de novo*, by recessive inheritance, or are merely sporadic events. Two groups have previously described one *MSH6* germline mutation each in two young CRC patients without family history of the disease (Chan *et al*, 1999; Verma *et al*, 1999). We describe six previously unreported *MSH6* genetic alterations in 38 early-onset CRC patients with negative family history of HNPCC-related cancer. Three of these six alterations are novel *MSH6* germline mutations (three out of 38 mutations; 7.9%), namely, a deletion and two missense mutations, one of the latter occurring contiguously to a silent mutation (Figure 1).

The *MSH6* mutation p.G1186fsX1190 is in all likelihood disease causing, as it leads to a premature stop codon at position 1190 and the predicted truncated protein looses one of the two MSH2- and the Mg^2+^-binding domains (Kariola *et al*, 2002). In addition, the patient's tumour presented MSI-H phenotype only in mononucleotide markers, a feature reported to occur preferentially in tumours associated with *MSH6* germline mutations (Verma *et al*, 1999; Wijnen *et al*, 1999), and screening for *MLH1* and *MSH2* mutations was negative. On the other hand, neither the 54-year-old father (Figure 2) nor the 47-year-old healthy blood donor carrying this mutation presented clinical symptoms of CRC (they had never performed colonoscopy screening), suggesting variable penetrance of this mutation. The modifier factors originating early-onset CRC in this 27-year-old patient remain unknown, but some data suggest that multiple mutations in different genes may influence the penetrance of the disease (Scheenstra *et al*, 2003; Soravia *et al*, 2005; Okkels *et al*, 2006). For instance, Okkels *et al* (2006) reported an 18-year-old CRC patient with three mutations (one missense *APC* mutation and a nonsense and a missense *MSH6* mutation), whereas none of the family members presenting only one or two of the three mutations presented colorectal neoplasms. Other authors have suggested that genetic and environmental factors may modify the risk conferred by mutations in cancer predisposition genes, as considerable inter-individual variation in age at cancer diagnosis has been observed in kindreds sharing the same genetic predisposition mutation (Heinimann *et al*, 1999; Bala and Peltomaki, 2001). Further studies are warranted to identify factors modulating the age of onset, penetrance, or tumour location in individuals with inherited MMR deficiency, as this knowledge may improve risk estimates and help identify individuals who are genetically susceptible to develop CRC at an early age.

The two missense mutations that were not detected in the normal population (Figure 1) result in a substitution of amino acids belonging to different polarity groups. The mutation p.R1321G occurred in a highly conserved region of the gene, the MSH2-binding domain, suggesting that it would affect MSH6 protein function. The p.[T216T; D217Y] mutation, although not located in a functional domain, results in the substitution of a negatively charged amino acid for a non-polar amino acid that may cause abnormal MSH6 protein function. As these two genomic alterations are likely to alter the function of the protein and were not found in the control population, it is likely that they are pathogenic mutations, but functional studies will be necessary to determine how each of these *MSH6* mutations affects protein function.

Besides the three presumably pathogenic mutations, we also identified three novel polymorphisms in the *MSH6* gene: the two silent mutations p.L758L and p.L773L and the intronic alteration c.3439-16C>T. On the other hand, the missense mutation p.V878A has previously been reported as a possible disease-causing mutation by some investigators (Wijnen *et al*, 1999; ICG-HNPCC database) and as a polymorphism by others (Peterlongo *et al*, 2003). Our finding of this alteration in the normal population corroborates that of Peterlongo *et al* (2003) and this change should now be classified as a polymorphism.

Of the 28 tumours that could be studied, MSI-H was found only in the case with *MSH6* deletion. This tumour shows instability only in mononucleotide repeats, that is consistent with the higher incidence of MSI in this kind of markers in MSH6-deficient tumours described in the literature (Verma *et al*, 1999; Wijnen *et al*, 1999). The MSI-L phenotype has also been related to germline *MSH6* mutations (Verma *et al*, 1999; Wijnen *et al*, 1999), but none of the two MSI-L tumours in this series showed *MSH6* mutations or *MLH1* promoter hypermethylation. The biological basis of the MSI-L phenotype is currently under debate. This phenotype could result from alterations in MMR proteins other than MSH2 and MLH1, from defects in genes not directly involved in MMR, or it could simply represent a ‘background’ level of genetic instability that may be detectable in all tumours if a sufficient number of markers is analysed (Lengauer *et al*, 1998).

We conclude that germline *MSH6* mutations contribute to a subset of early-onset CRC patients without a family history of the disease. Further studies are warranted to understand the genetic and environmental factors responsible for the variable penetration of these germline mutations and to identify other causes of early-onset CRC, as this would help genetic counselling of these patients and their relatives.

## Figures and Tables

**Table 1:** Clinicopathologic features of the 38 early-onset CRC patients

**Patient**	**Gender**	**Age at diagnosis of CRC (years)**	**Tumour location**	**Family history of cancer**
1	F	17	Ascending colon	—
2	M	27	Ascending colon	—
3	M	30	Rectum/sigmoid colon	—
4	M	33	Sigmoid colon	—
5	F	31	Sigmoid colon	—
6	F	42	Rectum	—
7	F	42	Rectum	—
8	F	34	Rectum	Father: lung cancer
9	M	19	Rectum	—
10	F	40	Rectum	—
11	F	38	Sigmoid colon	—
12	M	36	Rectum	Father: lung cancer
13	F	40	Rectum	—
14	F	30	Rectum	—
15	F	45	Rectum	—
16	M	39	Rectum	—
17	F	43	Rectum	—
18	F	45	Sigmoid colon	—
19	M	39	Rectum	—
20	M	45	Rectum	—
21	M	45	Ascending colon	—
22	M	31	Rectum	—
23	M	42	Rectum	—
24	M	38	Ascending colon	—
25	F	40	Rectum	—
26	F	32	Ascending colon	—
27	M	35	Rectum	—
28	M	35	Rectum	—
29	M	29	Sigmoid colon	—
30	M	31	Rectum	—
31	F	34	Rectum	—
32	F	39	Rectum	—
33	M	30	Rectum	—
34	M	38	Sigmoid colon	—
35	F	18	Rectum	—
36	F	36	Rectum	—
37	F	45	Rectum	—
38	M	42	Rectum	—
39	F	45	Rectum/sigmoid colon	—

CRC=colorectal cancer; F=female; M=male.

**Table 2:** *MSH6* germline alterations (and respective MSI status) detected in 38 early-onset CRC patients and in healthy blood donors

**Patient**	**Exon/intron**	**Nucleotide change^a^**	**Predicted effect**	**MSI**	**Blood donors**
37	Exon 4	c.[648A>T; 649G>T]^b^	p.[T216T; D217Y]	n.av.	0/108
25	Exon 4	c.2272C>T^b^	p.L758L	n.av.	n.an.
27	Exon 4	c.2319C>A^b^	p.L773L	MSS	n.an.
31	Exon 4	c.2633T>C	p.V878A	n.av.	2/108
15	Intron 5	c.3439-16C>T^b^	?	MSS	1/108
2	Exon 7	c.3558_3565delTGAAAGTA^b^	p.G1186fsX1190	MSI-H	1/108
14	Exon 9	c.3961A>G^b^	p.R1321G	n.av.	0/108

CRC=colorectal cancer; MSI=microsatellite instability; MSI-H=high-frequency microsatellite instability; MSS=microsatellite stable; n.an.=not analysed; n.av.=not available.

a According to GenBank accession no. NM_000179, nucleotide numbering starts with the A of the start codon.

b Not described in the literature.
